# Local entrainment of oscillatory activity induced by direct brain stimulation in humans

**DOI:** 10.1038/srep41908

**Published:** 2017-03-03

**Authors:** Julià L. Amengual, Marine Vernet, Claude Adam, Antoni Valero-Cabré

**Affiliations:** 1 CNRS UMR 7225, Institut du Cerveau et de la Moelle Epinière, Cerebral Dynamics, Plasticity and Rehabilitaion Group, Frontlab, Paris, France; 2 Epilepsy Unit, Dept. of Neurology, Pitié-Salpêtrière Hospital, APHP, Paris, France; 3 Department of Anatomy and Neurobiology, Laboratory of Cerebral Dynamics, Boston University School of Medicine, Boston, MA, USA; 4 Cognitive Neuroscience and Information Technology Research Program, Open University of Catalonia (UOC), Barcelona, Spain

## Abstract

In a quest for direct evidence of oscillation entrainment, we analyzed intracerebral electroencephalographic recordings obtained during intracranial electrical stimulation in a cohort of three medication-resistant epilepsy patients tested pre-surgically. Spectral analyses of non-epileptogenic cerebral sites stimulated directly with high frequency electrical bursts yielded episodic local enhancements of frequency-specific rhythmic activity, phase-locked to each individual pulse. These outcomes reveal an entrainment of physiological oscillatory activity within a frequency band dictated by the rhythm of the stimulation source. Our results support future uses of rhythmic stimulation to elucidate the causal contributions of synchrony to specific aspects of human cognition and to further develop the therapeutic manipulation of dysfunctional rhythmic activity subtending the symptoms of some neuropsychiatric conditions.

Brain rhythms subtend communication between neural assemblies^1^,^2^. Several lines of evidence have shown that the frequency^3^,^4^ and/or the phase^5^ of local and interregional oscillatory activity signal the engagement of different cognitive operations, influencing the emergence of specific behaviors. Moreover, relevant neurological deficits, impacting motor^6^, visuo-spatial^7^ and memory functions^8^,^9^,^10^ have been linked to the breakdown of neural oscillatory activity operating at specific frequency bands within and across cortical and subcortical brain sites. These reports have spurred a growing interest in the manipulation of human cerebral rhythms to better understand their causal physiological role in the coding of cognitive operations, and to treat some of the symptoms of neuropsychiatric disorders associated to dysfunctions of neural synchrony.

Non-invasive brain stimulation approaches, such as Transcranial Magnetic Stimulation (TMS) or transcranial Direct Current Stimulation (tDCS), have been employed for nearly two decades to modulate brain activity, showing promise in the treatment of neuropsychiatric conditions^11^,^12^. Applied at different stimulation parameters and configurations (e.g., adjusting frequency or inter-burst interval for TMS, and electrode polarity for tDCS applications), these technologies have the ability to induce concurrent (on-line) or long-lasting (off-line) modulations of excitability (increases or decreases) in circumscribed cortical regions, and spread such effects across brain networks following specific white matter connection pathways^13^,^14^,^15^. Whereas the on-line impact of TMS is thought to modulate brain processing and human behaviors by adding noise to ongoing neuronal communication (hence directly interfering with their coding strategies)^16^, its off-line effects are likely underlain by mechanisms of long-term potentiation (LTP) and long-term depression (LTD)-like plasticity, subtending longer lasting modulations of cortical excitability^17^. Notwithstanding, growing evidence on the role of oscillations (power, frequency and phase) in neural coding subtending the engagement of specific cognitive operations^5^ calls for a reinterpretation of the above-mentioned neurostimulation effects (particularly those induced by rhythmically repeated stimulation patterns). Beyond merely either interfering, suppressing or enhancing local brain activity, recent evidence supports the ability of rhythmic TMS stimulation to entrain local brain rhythms and oscillations with causal bearing on specific human cognitive processes and behaviors^18^,^19^,^20^.

To this regard, studies using rhythmic TMS and transcranial Alternating Current Stimulation (tACS), a stimulation modality employing a periodically varying direct current, combined with scalp EEG^21^ and Magnetoencephalography (MEG)^22^ recordings have contributed to ignite an interest in causally exploring the electrophysiological underpinnings of oscillatory entrainment. Moreover, these same approaches have proven extremely valuable tools to manipulate non-invasively rhythmic activity, showing for the first time convincing signs of entrainment in the human brain and demonstrating phase-, frequency-, site- and task-dependent impacts over cortical activity^18^,^23^ and cognitive processes such as visual perception^13^,^24^,^25^, visuo-spatial attention^26^ and memory consolidation^27^.

Notwithstanding, the poor focality provided by non-invasive stimulation technologies (particularly for tACS and to a minor extend also TMS) which can be estimated using computational models but not easily measured directly by monitoring changes in brain activity entrained during the delivery of stimulation^28^,^29^ and also the limited spatial resolution of non-invasive scalp recordings (particularly for EEG and to a minor extend also MEG) which do not always allow a precise localization of the entrained oscillatory activity as emerging from specific brain sites^30^, limit their ability to explore in further detail the physiology of oscillatory entrainment in well-circumscribed brain sites.

At the expense of losing the significant advantage of non-invasiveness for exploratory and clinical human applications, intracranial brain stimulation coupled to intracranial EEG recordings (iEEG) allows measuring the local effects of frequency-tuned direct electrical stimulation with a high degree of spatial certainty, which could be in a near future associated with the cytoarchitectural properties of the stimulated tissue and its precise connectivity patterns. To this regard, an existing clinical model that may facilitate an intracranial exploration of local neural entrainment in awake human systems is the one provided by pre-surgical mapping of medication-resistant implanted human epilepsy patients^31^,^32^. These clinical populations have their brain activity monitored in order to identify and localize the epileptogenic loci (i.e., brain regions triggering epileptic seizures) to be eventually removed surgically thereafter. Patients are implanted with arrays of intracerebral electrodes for a few weeks to pinpoint the localization of the seizing sources requiring removal and limit unwanted collateral damage during surgery. To avoid the risks of relying on spontaneous or behaviorally activated seizures, the same multielectrode arrays are often used in clinically controlled settings to deliver highly focal patterns of direct electrical stimulation at specific frequencies and intensities^33^,^34^, and survey causally the sensitivity of each implanted regions to generate seizures. Such enhanced causal mapping methods contribute to the identification and characterization of local epileptogenic activity evoked the electrical stimulation at a given frequency and intensity. Since the short-lasting electrical stimulation bursts applied to local neural assemblies (which are routinely used to survey epileptic and non-epileptic regions) contain frequency components, they allow for a direct causal study of neural entrainment in the human brain. Additionally, the use of iEEG recordings performed directly from brain sources reduces smearing effects present in scalp EEG signals mainly produced by conduction of the cerebrospinal fluids and extra-cerebral tissues such as arachnoid mater, dura matter, skull bone and other subcutaneous layers^30^.

To date, few studies have used direct electrical stimulation of the human brain with frequency-specific bursts of electric pulses^35^,^36^. However, these reports focused on the characterization of locally evoked activity, and neither analyzed nor reported any evidence supporting entrainment of oscillatory activity driven by the rhythm of an input source of stimulation. Hence, aiming to supplement prior evidence on entrainment mainly provided with non-invasive methods, and obtain evidence in support of focal oscillatory entrainment in humans directly from well-circumscribed brain regions, we analyzed iEEG recordings from sets of multi-electrodes implanted in a group of three epilepsy patients who received focal intracranial stimulation patterns performed during an awake causal mapping procedure aiming to localize and characterize epileptogenic cerebral loci, prior to considering their surgical removal.

## Results

**Figure 1:**
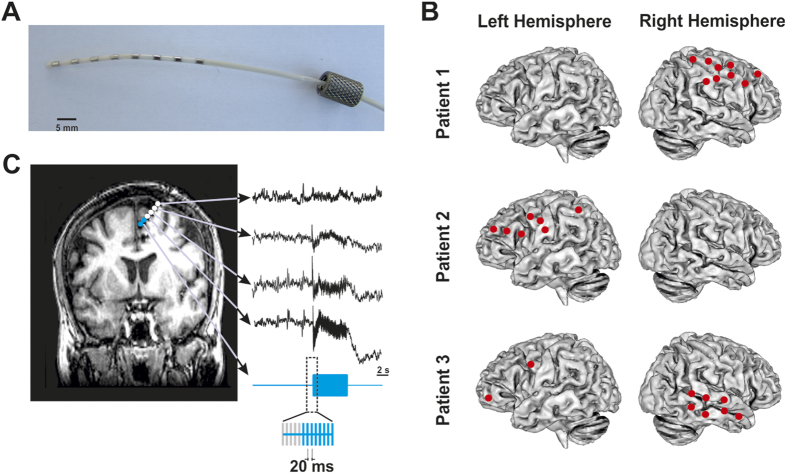
Multielectrode implantations and intracranial stimulation procedures. (**A**) Image of an 8-contact intracranial multielectrode employed for stimulation and iEEG recordings. (**B**) Diagram of the implantation sites of every individual multielectrode in the brain of the three patients considered in the analyses. (**C**) Detailed caption of a single multielectrode implanted in the frontal lobe of one of these patients, displayed on a T1 MRI scan (coronal view) recorded following implantation. Blue dots represent pairs of electrode contacts delivering 5 seconds long 50 Hz electrical bursts (blue iEEG trace). White dots signal the location of remaining contacts within the same electrode, recording brain iEEG activity concurrently with electrical stimulation patterns.

**Figure 2:**
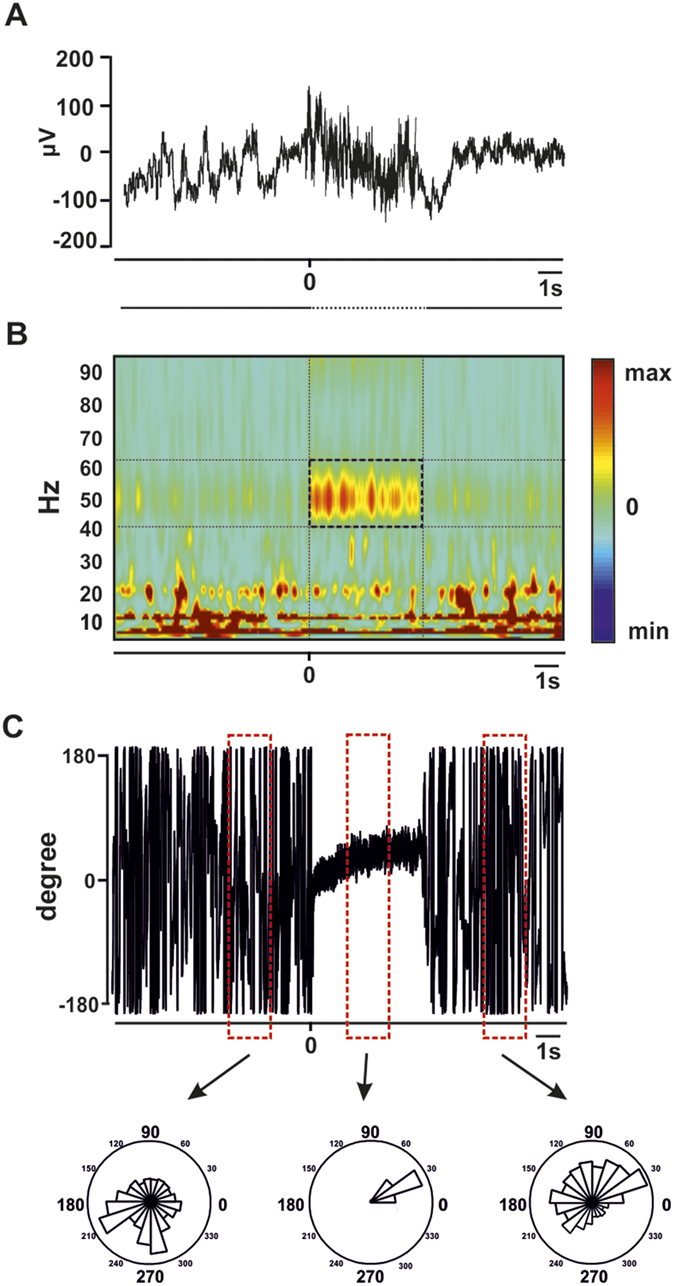
Enhancement of gamma oscilations time-locked to the stimulation input. (**A**) Representative iEEG trace from patient 2 displaying activity recorded from a multielectrode’s contact (multi-electrode 3, contact 2 located in the left superior frontal region) during a single 50 Hz stimulation burst delivered by a pair of contacts (contacts no. 5 and 6) hosted in the same multielectrode. The 0 ms time set corresponds to the onset of electrical stimulation. The black dotted line below depicts the duration of the stimulation burst. (**B**) Time-frequency analyses on this same single iEEG time-series reveal either increases (warm hues) or decreases (cold hues) of synchronization at each frequency bin, as compared to a baseline epoch, i.e., [−300 to −100 ms] recorded prior to the onset of electrical stimulation bursts. Note the increases of gamma synchronization (45–55 Hz) during the 50 Hz stimulation patterns. (**C**) (Top) Time series displaying fluctuations of the relative phase between the 50 Hz component of the iEEG time series plotted in (**A**) and the stimulator output signal. Angle phase histograms represent the distribution of relative phase values calculated during three two-second long epochs (red dotted boxes).

**Figure 3:**
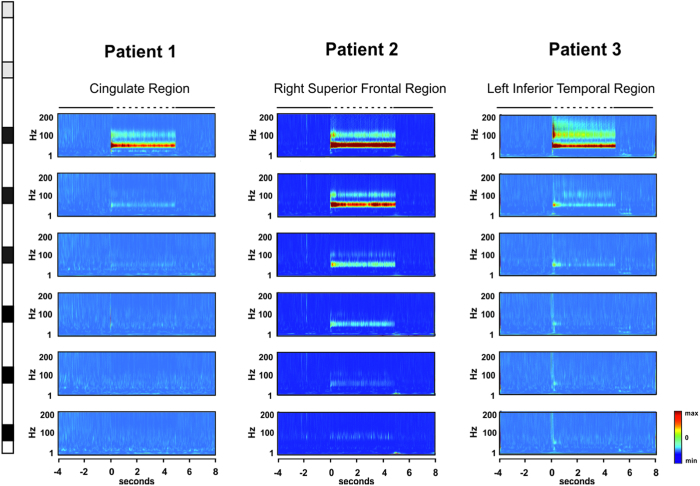
Time-Frequency activity maps for each individual patient included in the study. Representation of the time-frequency maps corresponding to the activity evoked by 3 different stimulations (one for each patient delivered through two adjacent contacts (shown in gray on the schematic representation of an intracranial multielectrode) and recorded by each of the remaining contacts (shown in black). The map shows increases (warm hues) or decreases (cold hues) of power for each frequency bin as compared to a baseline epoch, i.e., [−300 to −100 ms] recorded prior to the onset of electrical stimulation. Note increases in gamma synchronization (45–55 Hz) and associated harmonics (100 Hz) during 50 Hz stimulation patterns. The “0” ms time set corresponds to the onset of electrical stimulation. The dotted line shown above the top panel signals the duration of the 50 Hz stimulation burst.

We analyzed a total of 1059 iEEG epochs recorded during the delivery of intracranial stimulation at different intensities that ranged between 0.5–2 mA. These iEEG signals were recorded from cerebral regions characterized as non-epileptogenic on the basis of electrophysiological and clinical criteria. Figure 1 shows the stimulation and recording procedures, as well as the schematic representation of the implantations performed on each patient. Figure 2 illustrates the methodological approach for oscillatory entrainment analysis employed in our study. We found that 893 of these recordings (i.e., 84%) showcased increases of gamma (45 to 55 Hz) power during 50 Hz electrical stimulation bursts, fulfilling the first of the two conditions for oscillatory entrainment (See Methods section). Figure 3 displays, for each individual subject, a series of time frequency maps displaying changes in the power of oscillatory activity recorded by each of the contacts of a single multi-electrode prior, during and following 50 Hz bursts.

**Figure 4:**
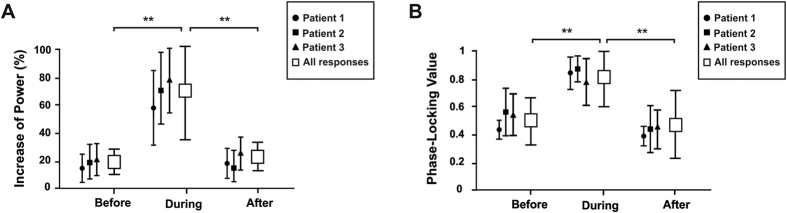
Increases of gamma power and phase-locking value during the stimulation period. Quantification of the individual single trial 50 Hz power (**A**) and phase-locking values (S-PLV) at this frequency band (**B**) for each of the three subjects of the study and group average values for these measures across stimulation trials and patients (white boxes) before, during and after the delivery of 50 Hz stimulation patterns. Power is here calculated as percent change relative to its value during the pre-stimulation period ([−300 to −100 ms]). The S-PLV index measures the stability of the phase difference (see Fig. 2C) ranging from 0 (random behavior) to 1 (constant phase syncrhony). Notice individual and group average increases of gamma (~50 Hz) power and S-PLV values ocurring specifically during the delivery of the stimulation patterns, which proved significantly lower before and after stimulation, as compared to during the delivery of 50 Hz electrical bursts (**p < 0.001 vs. before or after the stimulation burst).

**Figure 5:**
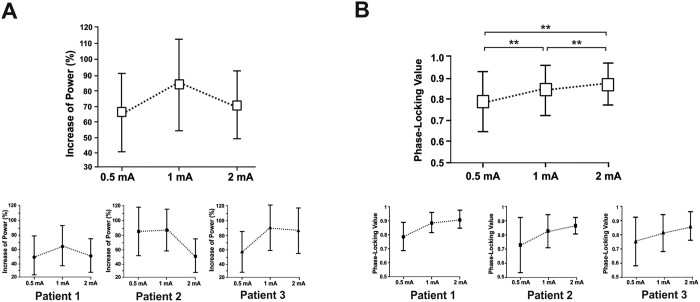
Dose-response curves for power and phase-locking value during the stimulation period. (Top panel) Group average power (**A**) and stability of phase-locking values (S-PLV) (**B**) for the three stimulation intensities considered in our analyses (0.5 mA, 1 mA and 2 mA) (white boxes), during 50 Hz stimulation. Power is calculated as the percent change relative to its values during the pre-stimulation epoch ([−300 to −100 ms]). The S-PLV index measures the stability of the phase difference (see Fig. 2C) ranging from 0 (random behavior) to 1 (constant phase synchrony). (Bottom panel) Individual dose-response curves of each of the three patients for power and the stability of the phase-locking value (S-PLV). Note that the S-PLV values increased significatly with accruing levels of stimulation intensity (**p < 0.001 vs. prior or following the 50 Hz stimulation period).

Differences between the increase of [45–55 Hz] power during 50 Hz stimulation period and the increase of [45–55 Hz] power during two other periods, one prior to the onset and the other following the offset of 50 Hz patterns were tested using non-parametric Friedman’s test. A significant effect of time-period was found [*χ*^*2*^(2, N = 893) = 1040, *p* < 0.0001]. Importantly, this effect was also present when this analysis was conducted individually on each patient [patient 1: *χ*^*2*^(2, N = 309) = 279.7, *p* < 0.0001; patient 2: *χ*^*2*^(2, N = 269) = 67.06, *p* < 0.0001; patient 3: *χ*^*2*^(2, N = 315) = 385.7, *p* < 0.0001]. Three post-hoc comparisons tested using Wilcoxon signed-rank test revealed significantly higher increases of [45–55 Hz] power during the 50 Hz stimulation burst (M = 74.06%, SD = 23.2) compared to either prior (M = 20.7%, SD = 5.6) or following (M = 22.03%, SD = 7.6) the 50 Hz stimulation period [*W* > 365462, *p* < 0.0001 for both comparisons; |*W*| > 38830, *p* < 0.0001 for these comparisons in each of the three patients] (See Fig. 4A). No difference was found when comparing the increase of [45–55 Hz] power prior and following the stimulation [*W* = 18554, *p *= 0.18]. The impact of stimulation intensity on the level of power increases at [45–55 Hz] during 50 Hz patterns was tested using the Kruskal-Wallis test. This test did not reveal any effect of intensity in the increase of [45–55 Hz] power [*H* = 3.78, *p* = 0.15]. (Figure 5A shows dose-response curves for power).

A comparison of the mean S-PLV at [45–55 Hz] during the 50 Hz stimulation period with the two other periods, one prior to the onset of 50 Hz stimulation and another following the offset of 50 Hz stimulation was performed using non-parametric Friedman’s test. A significant effect of time-period [*χ*^*2*^(2, N = 879) = 855.5, *p* < 0.0001] was found after correcting for surrogate data (see Methods Section and Supplementary Materials, for details on how surrogate data were generated and how the correction was tested). Only 1.2% (i.e., n = 14) of the iEEG traces considered for S-PLV analysis did not pass the surrogate test and were excluded from the statistical analysis. Similarly to the analysis of power described above, statistical differences in S-PLV were also found individually in each of the three patients [patient 1: *χ*^*2*^(2, N = 305) = 323.6, *p* < 0.0001; patient 2: *χ*^*2*^(2, N = 264) = 327.1, *p* < 0.0001; patient 3: *χ*^*2*^(2, N = 310) = 467.7, *p < *0.0001]. Three post-hoc comparisons were tested using the Wilcoxon signed-rank test. These post-hoc analysis revealed significantly higher increase of S-PLV during the 50 Hz stimulation burst (M = 0.80, SD = 0.15) compared to either prior (M = 0.52, SD = 0.13) or following (M = 0.48, SD = 0.18) the stimulation period [*W* > 430252, *p* < 0.0001 for both comparisons; |*W*| > 13940, *p* < 0.0001 for these comparisons in each patient] (Fig. 4B). No difference was found comparing the S-PLV prior and following 50 Hz stimulation [*W* = 26728, *p *= 0.101]. The impact of the intensity on the level of S-PLV during stimulation was tested using the Kruskal-Wallis test. Interestingly, an effect of intensity of stimulation was found [*H* = 17.06, *p* = 0.0002]. Post-hoc comparisons using Mann-Whitney test showed that S-PLV increased marginally from 0.5 mA to 1 mA [S-PLV at 0.5 mA vs. S-PLV at 1 mA *U* = 38061, *p* = 0.06] and significantly from 1 mA to 2 mA [S-PLV at 1 mA vs. S-PLV at 2 mA, *U* = 44481, *p* < 0.0001] (Fig. 5B shows dose-response curves for S-PLV). Importantly, such entrained rhythmic activity was not time-locked to any other frequency band than the one provided by 50 Hz electrical patterns, neither present prior or following the stimulation bursts.

Interestingly, we found that the S-PLV of the relative phase between the stimulation source and 50 Hz iEEG entrained activity correlated inversely with the distance (in mm) between recording and stimulation electrode contacts (Spearman rank-correlation, *r*_*s*_ = −0.15, *p* < 0.0001). Additionally, we found that this relative phase positively correlated with the distance (in mm) between the recording and stimulation electrode contacts (Spearman rank-correlation, *r*_*s*_ = 0.22, *p* < 0.001).

In sum, phase-synchronization for entrained gamma activity occurred only during the delivery of 50 Hz stimulation, suggesting an episodic phase-alignment of this rhythm to the frequency of the electrical burst generated by the electrical stimulation source (Fig. 2C) hence fulfilling the second requirement characterizing oscillatory entrainment.

## Discussion

A growing body of studies supports the modulation of specific human behaviors during the non-invasive manipulation of local oscillatory activity, either by entraining or by interfering the buildup of specific brain rhythms^19^,^24^,^27^,^37^,^38^,^39^,^40^. In such framework, the use of phase- or frequency-tuned rhythmic sources of activity to manipulate local oscillations and interregional synchrony is gaining momentum as an approach to causally confirm suspected links between normal/pathological oscillatory activity and brain function/dysfunction and also to drive cognitive improvements or rehabilitate faulty cognition in neuropyschiatric patients (see refs 42 and 43 for review).

Extremely valuable pioneering contributions employing TMS^18^,^43^ or tACS^44^ stimulation methods concurrently with scalp EEG and MEG^22^ recordings have provided consistent evidence supporting the entrainment of frequency-specific oscillations in awake humans and triggered renewed interest in the electrophysiological underpinnings of such phenomena. Nonetheless, limitations in spatial resolution of non-invasive stimulation methods^45^, the uncertainty with regards to the distribution of the electrical currents^21^, compromised signal-to-noise ratio under the influence of TMS/tACS generated electrical fields and localization power of scalp recording methods^46^ calls for supplementary evidence that completes a convincing demonstration of oscillatory entrainment phenomena in well-circumscribed regions of the human brain and further studies on their underlying features and mechanisms.

In the present study, we studied such phenomenon in datasets from clinically-guided causal mapping sessions performed in intracranially implanted epilepsy patients. Invasive intracranial stimulation and recording methods measure a more direct neurophysiological signature of electrically induced local oscillatory entrainment, supplementing prior evidence, which might facilitate the study of its neurophysiological underpinnings. We showed that 50 Hz electrical bursts entrained neural oscillations at a specific frequency dictated by the output rhythm of the stimulator. These effects were induced during the delivery of the stimulation bursts, wearing off rapidly as soon as the electrical source was discontinued, they were observed at a single-trial level, and proved highly consistent across individual participants and stimulation trials. Importantly, the above-mentioned phase-difference shifts between the stimulation and the iEEG time series as a function of the distance between the two contacts delivering the electrical current and the contact recording iEEG signals, and the varying internal dynamics shown by phase-locking measures (such as phase difference and S-PLV) during the electrical burst rule out the confounding of mass conduction artifacts (which would equally impact recordings across contacts and would fail to display internal dynamics emerging from interactions between entrained and ongoing natural oscillations) and support the physiological nature of the stimulation-entrained oscillations reported in our study.

The study of these phenomena in datasets obtained during clinically-guided causal mapping sessions in intracranially implanted epilepsy patients poses some limitations. Very importantly, the variety of implantation schemes, the low number of stimulation parameters (frequency, duration), and the scarce number of samples per condition (combining a given site and stimulation intensity) that can be tested to keep mapping sessions within a reasonable duration, avoiding an overstimulation of the cerebral tissue, remain a limitation of this approach. Notwithstanding, we benefited from the high effectiveness and focality of these approaches applied to well anatomically characterized cerebral sites, combined with the unique localization power and the outstanding signal-to-noise ratio provided by human iEEG recordings^33^.

Two additional limitations of this study deserve special discussion. The first one is the difficulty to disentangle whether the oscillatory activity observed during 50 Hz stimulation reflects a physiological response to the stimulation itself or is rather an effect of strong electrical artifacts recorded or generated during the analyses. A potential source of artifacts in our data could emerge from the artifact removal procedure used to eliminate undesirable noise tied to the electrical pulses. This method, which has been already used and validated in studies analyzing evoked activity induced by single intracranial electrical pulses^34^,^47^,^48^ consists in the interpolation of the removed artifacted period using a third degree spline. We tested the ability of this approach to remove any contributions from oscillatory activity at the frequency of interest (50 Hz) originated from a non-physiological source, by conducting a series of statistical control analysis using real and artificially artifacted datasets. Our analyses (see Supplementary Materials, Figures S1 and S2) demonstrate that this procedure was efficient in removing undesirable contributions of 50 Hz activity in the signal that might potentially explain our results. A second potential confounding could be the recently reported non-linear modulations of the stimulation artifact^49^ in scalp EEG under tACS stimulation, likely induced by rhythmic changes of the body’s impedance and head and body motion driven by the activity of the heart and breathing which given their complex nature and frequency could be misinterpreted as neural entrainment. The lack of electrocardiogram and breathing recording performed along prevents us to directly address this eventuality in our own data. Nevertheless, no signs of artifacts similar to those reported by Noury and colleagues^49^ were revealed by our analyses, and to our knowledge, no study has yet reported that similar artifacts might equally impact concurrent intracranial recordings performed during direct electrical stimulation of brain tissue. Moreover, the recording contacts of intracranial electrodes are in direct contact with brain tissue, ensuring a lower and more stable impedance values than those of scalp electrodes^50^, a fact that would potentially reduce the sensitivity of our recording to such non-linear phenomena. Last but not least, Noury and colleagues^49^ reported the effects of such non-linear contributions to power but not to phase locking value, which remains the main outcome measure analyzed in our manuscript (heartbeats and breathing motion are randomly positioned in relation to phase of the stimulation and therefore such artifacts could not be locked to the stimulation pattern as the entrainment responses we report in our manuscript).

A second limitation of this study is that stimulation was delivered at the same frequency of the line-noise, a fact that may limit an accurate interpretation of our findings which rather than showing an induced physiological effect, may reflect an interaction with line-noise. However, our analyses strongly suggest that this last scenario is unlikely. First of all, we found statistically significant increases of gamma power ([45–55 Hz]) during the stimulation period, as compared to periods prior and following the stimulation (all three periods were compared against the same pre-stimulation interval used as a baseline). Second, even if there was phase coupling prior and following the 50 Hz stimulation bursts (presumably because line-noise has a similar temporal structure than the delivered burst), this measure was significantly higher during the delivery of the pulses, suggesting that such modulation in S-PLV cannot be solely explained as an artifact generated by 50 Hz line-noise. Third and last, additional tests performed on either artificially artifacted vs. real iEEG data included in the manuscript (see Supplementary Materials) suggest that the method used to remove the artifact produced by the stimulation, if anything, tends to reduce the contribution of 50 Hz stimulation towards line-noise values. In sum, although we cannot completely rule out the presence of 50 Hz line-noise in our data, we suggest that the nature of the effects of the stimulation reported in this study is mainly physiological.

An intriguing result of our study is the lack of regional-specificity of the physiological response to 50 Hz stimulation. To this regard, and according to TMS studies on natural frequencies^35^, we would expect local gamma entrainment to be more likely in areas in which this is the ongoing “natural” oscillation frequency (see criteria for establishing neural entrainment that is listed by Thut and coworkers^51^). A more detailed analysis of region-specific reactions to gamma (50 Hz) entrainment will require accruing and analyzing in a common anatomical space, data from larger cohorts of patients. In attendance of accruing enough data to reliably address this issue, our study suggests the possibility of gamma entrainment phenomena in brain regions within the frontal, parietal and temporal lobe, hence, quite irrespective of the targeted anatomical region. Two non-mutually excluding hypotheses could explain this unexpected finding. First, since gamma rhythms are widely distributed across brain systems^52^ and contribute to many cognitive domains (e.g. attention^53^,^54^, perception^55^,^56^, language^57^,^58^ and memory^59^,^60^), 50 Hz oscillatory entrainment with direct intracranial stimulation might be easy to elicit in many areas of the human brain. Second, we could also entertain the hypothesis that at difference with noninvasive stimulation methods^22^,^43^ the highly focal and the very intense stimulation source provided by intracranial electrical stimulation has the potential to impose fast rhythms outside the boundaries of the “natural” frequency characterizing a given site. In support of the latter possibility, evidence from animal studies using frequency-tuned optogenetic stimulation^61^,^62^, also support entrainment of neural oscillations regardless of the “natural” frequency operating in the stimulated region. The discussion of this point opens a fair debate on whether or not entrainment in a given area would be particularly facilitated when stimulation is tuned to the most “natural” frequency operating on the stimulated site. An interesting approach to test this claim would be to compare in the same implanted region the likelihood and/or level of entrainment induced by different stimulation frequencies. Since the current study is based on existing iEEG datasets obtained following strict clinical criteria, hence using only 50 Hz bursts (the clinical state-of the-art frequency maximizing the likelihood of inducing seizures^63^), we are unable at this time to test this hypothesis. Future studies will be directed to further characterize crucial features associated to oscillatory entrainment phenomena, in particular their local vs. network distributed effects, their dependence from local natural rhythms and cytoarchitecture and the detailed influence of ongoing rhythmic activity present in the targeted region prior to stimulation.

In conclusion, by combining intracranial stimulation with specific behavioral paradigms, our approach has the potential to open new venues to explore the causal links between brain oscillation rhythms and cognition in humans. To this regard, understanding the neurophysiological basis of oscillatory entrainment with direct brain stimulation methods holds the potential to guide an efficient use of invasive or non-invasive approaches to manipulate abnormal oscillatory activity subtending specific neuropsychiatric disabilities and contribute to their rehabilitation.

## Methods

### Patients

The iEEG datasets of three medication-resistant epilepsy patients (2 females and 1 male, respectively 29, 32 and 25 years old) obtained during direct intracranial stimulation performed in the context of clinically guided causal mapping sessions, aiming to causally identify epileptic foci, prior to considering the feasibility of surgical removal^31^,^32^, were included in our analyses. Two patients were implanted with 9 multi-electrodes each, whereas a third one received 7 multi-electrodes. Patients provided an informed consent, and recordings were performed under ethical committee agreement. Implantation sites were planed and selected exclusively on purely clinical criteria, with no reference to the present analysis. The study was sponsored by the INSERM and approved by the ethical committee (CPPRB, Comité Consultatif de Protection des Personnes participant à une Recherche Biomédicale) Ile de France I (reference number C11–16, 5–04), which regulates the publication of anonymized versions of participant’s data. All methods were performed in accordance with the National (France) and International (European Union) guidelines and regulations, and with the Declaration of Helsinki.

### Multielectrode implantations, recordings and intracranial stimulation procedure

Intracerebral multi-electrodes provided with 6 to 10 contacts (Adtech, Racine, Wisconsin, USA) were used in concurrent stimulation and iEEG recordings (See Fig. 1A). A detailed account of the number of multi-electrodes, their number of contacts and the covered cerebral regions on each patient is presented in Table 1 and Fig. 1B. Depending on each electrode type and model, contacts were 2–3 mm long, 1 mm diameter and spaced 5 mm apart from each other. The implantation procedure was guided with a Leksell stereotactic frame (Elekta, Stockholm, Sweden) and sequences of T1 magnetic resonance imaging (3T, General electric, Fairfield, Connecticut) performed prior and following the implantations were used to plan the procedure and verify the location of each multi-electrode on each patient’s brain, respectively.

Electrical stimulation was delivered through a programmable clinical Micromed stimulator allowing the design of complex electrical waveforms. The standardized clinical stimulation protocol consisted in the application of 5-seconds electrical bursts of 250 biphasic squared pulses (1 ms pulse width), delivered every 20 ms (i.e., at a 50 Hz frequency). Stimulation was systematically delivered by spatially adjacent pairs of contacts, progressing either from the deepest to the most superficial contacts or vice-versa upon decision of an expert neurologist in charge of the mapping session. The exclusive use of gamma stimulation at a 50 Hz frequency in causal mapping procedures on epileptic patients is based on clinical research demonstrating that this is the most likely frequency that induces an excitatory response followed by a self-sustained after-discharge and a clinical seizure^63^ in case an epileptogenic region is targeted. These same clinically standardized protocols aim to maximize the chances of causally identifying epileptic sites and minimize tissue damage by using the lowest possible number of electrical bursts and keeping testing sessions short. Bursts were delivered at increasing intensities, from 0.5 to 5 mA, through pairs of adjacent multi-electrode contacts at least 45 seconds apart (Fig. 1C). The multi-electrodes and stimulation intensities employed for each delivered stimulation were selected on a case-by-case basis, by an expert neurologist in charge, according to the patterns of iEEG activity and the clinical responses evoked by prior manipulations on each patient. Following these established clinical procedures, inter-burst intervals were randomized in duration but kept always longer than 45 seconds to avoid carry-over excitability effects across successive bursts^33^. Stimulation was delivered between pairs of adjacent contacts from the same multi-electrode while iEEG raw data were recorded from the remaining contacts. Data acquisition was performed using a 16 bits Micromed Amplifier system (Micromed, Mogliano Veneto, Italy). The sampling rate was set to 1024 Hz and during the acquisition, the signal was band-pass filtered at 0.15–350 Hz. An external electrode located on the scalp FCz position (10/20 EEG system) was used as a reference for the iEEG recordings. These recordings were obtained from each available contact on each of the implanted multi-electrodes. Nonetheless, only iEEG data from sites classified and logged post-hoc as non-epileptogenic by an expert neurologist according to iEEG and clinical criteria were considered for further analyses.

During intracranial stimulation sessions (direct brain stimulation), patients were fully awake and comfortably seated in a chair placed in a well-lighted room. Shortly before each of the stimulation bursts, patients were asked to remain relaxed and keep their gaze on a fixation cross located beyond arm’s reach in front of them. The duration of the stimulation session and the number of stimulation bursts received by each patient were determined following strict clinical criteria and managed by an expert neurologist in charge of the stimulation. Patient 1 underwent a session of ~6 hours in which she received 98 bursts of stimulation (23 bursts at 0.5 mA, 23 at 1 mA, 24 at 2 mA, 16 at 3 mA, 8 at 4 mA and 4 at 5 mA). The stimulation session in patient 2 lasted ~3 hours during which she received the amount of 46 bursts of stimulations (16 bursts at 0.5 mA, 19 at 1 mA, 6 at 2 mA, 2 at 3 mA, 2 at 4 mA and 1 at 5 mA). Finally, duration of the stimulation session performed on patient 3 lasted for ~5 hours and the patient received 92 stimulation bursts (19 bursts at 0.5 mA, 29 at 1 mA, 29 at 2 mA, 10 at 3 mA and 4 at 4 mA).

### iEEG data pre-processing

Intracranial EEG responses to electrical stimulations were recorded from the remaining sensors within the same multi-electrode prior, during and shortly after low intensity stimulation of non-epileptogenic cerebral sites (Figs 1A and 2A). To avoid the confounding of long stimulation artifacts, only trials carried out at stimulation intensities of 0.5 mA, 1 mA and 2 mA were considered for further analyses. For each stimulation trial, we analyzed 28 seconds of iEEG data (8 s prior and 20 s following the onset of the burst, hence including burst duration) recorded by contacts of the same multi-electrode not involved in the delivery of the stimulation. A pre-processing pipeline was designed using in-house software coded in Matlab (Mathworks, MA, USA). Pre-processing steps included procedures for artifact removal and reference signal subtraction. The stimulation induced a stereotyped artifact on iEEG signals from the recording contacts, which consisted in high-amplitude waveforms lasting about 8 ms after each electrical pulse. The 8-ms portion of the signal corresponding to the stimulation waveform was systematically removed and the iEEG-blank periods left in the recordings by this operation were interpolated using a weighted cubic spline method^47^,^64^. Procedure to assess the potential contamination produced by this artifact removal algorithm is described at the Supplementary Methods section.

A reference subtraction procedure was applied to curtail potential contamination of iEEG time series by the scalp reference signal and reduce their potential impact on phase-synchrony analyses^65^,^66^,^67^. The procedure estimated the reference signal from recorded signals using a method based on a Blind Source Separation (BSS)^67^. A reference-free signal for each of these contacts was obtained by adding the estimated reference signal from each iEEG recording.

### Time frequency maps and phase-coupling analysis

We defined the feature signature of electrically-induced rhythmic entrainment as an enhancement of frequency specific local oscillatory activity synchronized in phase (i.e., phase-locked) to the individual pulses integrating the source of rhythmic electrical stimulation^18^, lasting for the duration of the electrical bursts. Therefore, we considered that a reliable occurrence of oscillatory entrainment required an increase of power at 50 Hz, lasting across the stimulation period, and an enhanced and sustained phase-coupling of this induced rhythmic activity with the output of the stimulation, tightly restricted to the duration of the delivered electrical bursts^18^,^43^.

Time frequency maps were extracted by convoluting each reference-free iEEG signal with a complex Morlet wavelet^3^,^68^ (see equation (1)):


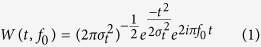


in which the relation 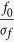 (were 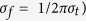 was set to 6.7^3^. We explored effects on frequencies from 1 to 200 Hz, at 1 Hz steps. For each stimulation event, we computed the time-varying power from each recording contact (Fig. 2B). We then calculated the percent change with respect to a baseline [−300 to −100 ms], determined prior to the onset of the first pulse of the burst for the 45–55 Hz frequency band.

To address the extent to which electrically induced gamma oscillations were time-locked to the electrical pulses delivered by the stimulator (therefore, evoked gamma oscillations), we calculated the temporal dynamics of the instantaneous relative phase between the electrical output of each burst and the 50 Hz-component of each of the iEEG time series recorded by contacts within the stimulating multielectrode. Only recordings from contacts showing average power increases in the [45–55 Hz] band during the stimulation period 20 times higher than the pre-stimulation onset baseline were considered in these analyses^69^. We computed for each time sample, the instantaneous phase difference between the gamma [45–55 Hz] iEEG components (post-artifact removal) recorded by each contact and a modeled signal emulating the 50 Hz pattern delivered by the electrical stimulator (Fig. 2C). In order to create a signal emulating the stimulation bursts, we implemented computational approaches with an in-house-programmed script in Matlab (Mathworks, MA, USA) to design and generate an artificial signal at the same sampling rate (1024 Hz) as the original data, with the value “+1” (followed by a “−1” to account for its biphasic waveform) repeated every 20 ms and a value of “0” everywhere else in the time series. This newly generated signal had the same length as each of the iEEG traces, and took the “+1” at the time point corresponding to the onset of the stimulation. These calculations allowed us to characterize phase-synchrony on the iEEG signals recorded by each individual multielectrode contact for the duration of the 50 Hz bursts, and most importantly to compare such values to those calculated prior and following electrical stimulation. Similar procedures have been used to measure the phase locking value between a source of stimulation and local evoked oscillatory activity in concurrent tACS-MEG studies^22^. To this end, we computed the angles of the Morlet wavelet coefficients of both signals using the equation (2):


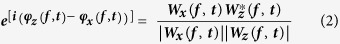


where W represents the Morlet coefficient for each frequency f and time t; x and z are each of the two signals (iEEG and the stimulator) and φ_x_ and φ_z_ their instantaneous phase^70^.

We calculated the consistency over time of the phase difference between these two signals by using the single-trial phase-locking value (S-PLV)^71^ described in the equation (3):


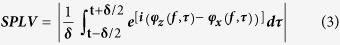


where δ represents a time-length covering a specific number cycles within the frequency of interest. Following published recommendations^70^,^72^, we used δ= 200 ms that corresponded to 10 cycles of the 50 Hz frequency of interest. This measure detects the stability of the phase difference during a single trial (i.e., considering it a single measurement) and its values range from 0 (signaling random fluctuations of phase-difference) to 1 (indicating the strongest phase-locking possible.

### Statistical Analyses

To demonstrate the emergence of 50 Hz oscillations locked to the 50 Hz electrical bursts during the stimulation period (as compared to epochs prior and following the stimulation), we tested for differences of increase of power at the stimulation frequency band (in the [45–55 Hz] band) and the S-PLV prior, during and following the delivery of the bursts using non-parametric Friedman’s test. For each stimulation burst delivered through a pair of active contacts, we considered the iEEG traces recorded from contacts within the same multi-electrode, not assigned to deliver the electrical current. Each single-trial value of power and S-PLV from each of the 3 patients was included in our analyses as an independent observation. Those values were consolidated as part of the same dataset and integrated in the same analysis to assess general effects of entrainment phenomena. These same values were also analyzed for each patient in order to assess consistency across individual participants. Analyses of power and S-PLV, were based on three different temporal windows introduced as dependent variables in the Friedman’s test (Time-period factor): [−4000 to −2000 ms] before 50 Hz electrical bursts onset (Power pre and S-PLV pre); [2000 to 4000 ms] after the 50 Hz burst onset (Power during and S-PLV during) and [8000 to 10000 ms] after the 50 Hz burst onset (Power post and S-PVL post: 3000 to 5000 ms after the 50 Hz burst offset). Post-hoc comparisons were tested using non-parametric Wilcoxon signed rank test.

A second analysis was performed to test whether or not there was a relationship between the intensity at which the electrical stimulation had been delivered (either 0.5 mA, 1 mA or 2 mA) and the values of power and S-PLV obtained during the 50 Hz stimulation burst. To this end power and S-PLV values during the stimulation period for each of these three intensities were included into a non-parametric Kruskal-Wallis test with factor Intensity (0.5 mA, 1 mA, 2 mA). Post-hoc comparisons were tested using the non-parametric Mann-Whitney test.

Since in our study S-PLV data analyses were single-trial based, we generated a large ensemble of surrogate time series (n = 30 for each trace) sharing some statistical features with the original data in order to create a null-distribution of S-PLV obtained from independent oscillators. This method allowed us to avoid false positive S-PLV during the stimulation, and consider only S-PLVs that were significantly different from what would be expected for independent oscillators (See Supplementary Materials). To this aim, for each iEEG trace, surrogates that preserved the power spectrum of the instantaneous frequency were created, randomizing the phase spectrum while preserving the amplitude spectrum^73^.

Non-parametric Spearman rank tests were used to estimate the statistical significance of the correlation between the distance (in mm) between recording multielectrode contacts and the stimulation contact pair, (i) and the relative phase between the stimulation output signal and the 50 Hz iEEG recorded components (ii) and the S-PLV value.

For all tests, significance level was set at *p* < 0.05. All *p* values obtained from post-hoc tests were corrected for multiple comparisons by means of the Bonferroni correction.

## Additional Information

**How to cite this article:** Amengual, J. L. *et al*. Local entrainment of oscillatory activity induced by direct brain stimulation in humans. *Sci. Rep.* **7**, 41908; doi: 10.1038/srep41908 (2017).

**Publisher's note:** Springer Nature remains neutral with regard to jurisdictional claims in published maps and institutional affiliations.

## Supplementary Material

Supplementary Methods

## Figures and Tables

**Table 1:** Summary table with demographic data (gender and age) of the three patients (P1, P2 and P3) included in our analyses, the number of implanted multielectrodes, the total number of contacts and the name of the cerebral regions covered by the implantations on each patient.

Patient reference	Gender	Age	Number of multielectrodes	Total number of contacts	Implanted regions
P1	F	29	9	54	Right medial frontal lobe
P2	F	32	7	48	Left medial frontal lobe Left parietal lobe
P3	M	25	9	78	Right temporal lobe Left orbitofrontal region Left superior frontal lobe

F: Female; M: Male.
