# Supplementary material for: Local entrainment of oscillatory activity induced by direct brain stimulation in humans
Source: Sci Rep. 2017 Mar 3;7:41908. doi: 10.1038/srep41908 (PMC5335652; doi:10.1038/srep41908)
Supplement: Supplementary Methods [file srep41908-s1.docx]

**Local entrainment of oscillatory activity induced by direct brain**

**stimulation in humans**

Julià L. Amengual ^1^#, Marine Vernet ^1^#, Claude Adam ^2^, Antoni Valero Cabré ^1,3,4*^

^1^ CNRS UMR 7225, Institut du Cerveau et de la Moelle Epinière, Cerebral Dynamics, Plasticity and Rehabilitaion Group, Frontlab, Paris, France; ^2^ Epilepsy Unit, Dept. of Neurology, Pitié-Salpêtrière Hospital, APHP, Paris, France; ^3^ Department of Anatomy and Neurobiology, Laboratory of Cerebral Dynamics, Boston University School of Medicine, Boston, MA, USA; ^4^ Cognitive Neuroscience and Information Technology Research Program, Open University of Catalonia (UOC), Barcelona, Spain. (#) Both authors contributed equally to this work.

**Supplementary Methods**

1. **Ability of our artifact removal procedure to cancel stimulation artifacts in iEEG signals**

We conducted a list of control experiments aimed to demonstrate the capacity of our method to remove stimulation artifacts produced by the effects of the 50 Hz electrical currents induced by the stimulation. To this end, we randomly selected 65 iEEG traces from different contacts across the three patients of the study. Each of these iEEG traces were chosen from contacts located the closest from the stimulation contacts (defined as the pair of contacts within the same multielectrode that delivered the 50 Hz electrical stimulation bursts) in order to get a clear picture of the artifact shape. These iEEG traces were 20 seconds long, centered to the onset of the stimulation, and contained iEEG signal during the delivery of electrical stimulation at 2 mA (the maximal stimulation intensity considered in our analyses). For each of these iEEG traces, we calculated the power spectral density (PSD, in V^2^/Hz) of 1 second epochs of iEEG signal for the following 5 conditions (see Figure S1):

1. PSD of iEEG signals recorded prior to the delivery of 50 Hz stimulation bursts.

2. PSD of iEEG signals recorded during the delivery of 50 Hz stimulation bursts

3. PSD of artificially artifacted iEEG signals. We selected 50 consecutive 8 ms long periods of iEEG signal during the stimulation period and we replaced these periods by individual artifact waveforms selected from the stimulation period to obtain *artificially artifacted* real iEEG traces.

4. PSD of artificially artifacted iEEG signals following our artifact removal procedure. We applied the artifact removal procedure employed in our study to the artificially artifacted signals obtained in condition 3*.*

5. PSD of the iEEG signals recorded during 50 Hz electrical stimulation bursts after artifact removal.


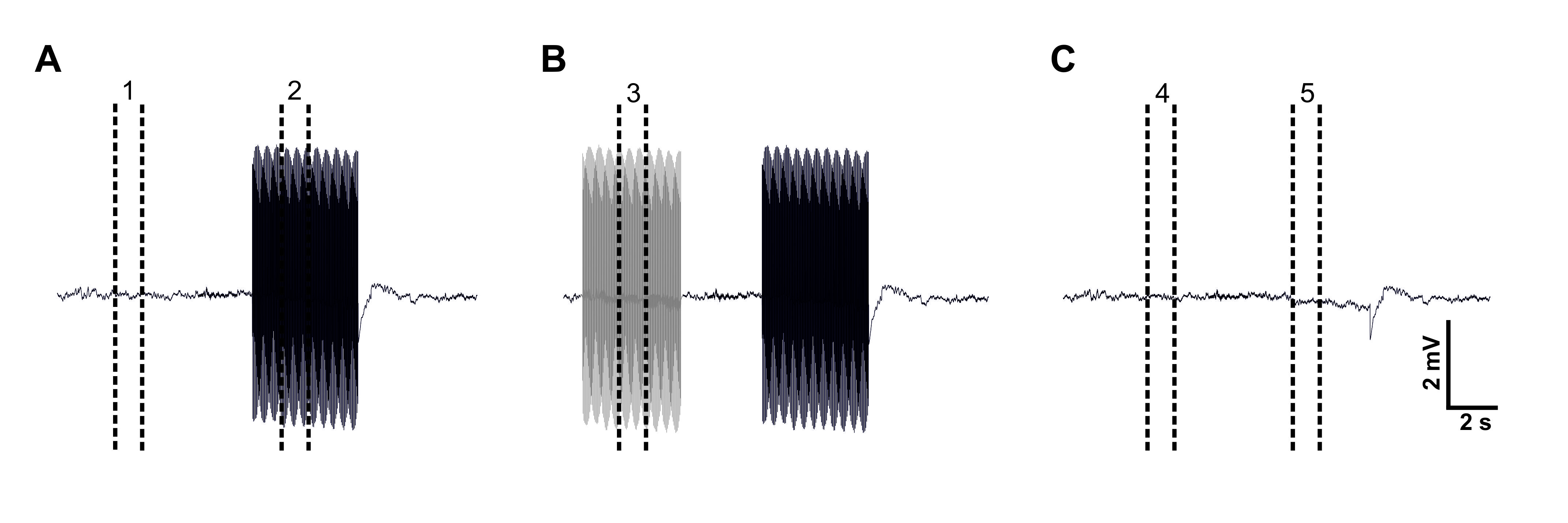


Figure S1 : (A) Representative iEEG trace recorded in a multielectrode contact adjacent to two active stimulation contacts, during the delivery of a 5 seconds burst of 50 Hz stimulation at an intensity of 2mA. (B) Shows the same iEEG trace presented in caption A with added stimulation artifacts to the pre-stimulation period (gray area). (C) The same iEEG trace presented in A after the application of the artifact removal procedure employed in our study. The number shown along the traces (see 1,2,3,4 & 5) label the iEEG periods in which the procedures defined in the five conditions of this control experiment were applied.

We first tested whether iEEG signals during the real stimulation and *artificially artifacted* iEEG signals with the same waveforms as those obtained from the artifacts recorded during stimulation had a similar amount of gamma power within a frequency band of interest [45-55 Hz] (see Figure S2 A). A Wilcoxon Signed-ranks Test indicates that the level of gamma power within a 45-55 Hz band was similar in both conditions [*W* = 398, *p* = 0.19].

Second, we measured the ability of our artifact removal method to cancel the gamma frequency component of the 50 Hz stimulation artifact. A Wilcoxon Signed-ranks Test indicated that the level of power within 45-55 Hz was significantly reduced [*W* = 2016, *p* <.0001] (Figure S1 B). In addition, we compared the level of ‘residual’ 50 Hz power activity after artifact removal in *artificially artifacted* iEEG signals, with the power of the line-noise captured by iEEG recordings. A Wilcoxon Signed-ranks Test indicated that the level of power within the 45-55 Hz gamma frequency band was not significantly different [*W* = 230, *p* = .44, see Fig S1 C] between these two condtions. This analysis shows that after applying our artifact removal procedure, iEEG signals contain similar levels of contamination by line-noise than the original recordings.

Third, we compared the amount of ‘residual’ 50 Hz oscillatory activity present after removing stimulation artifacts from *artificially artifacted iEEG* signals and in iEEG signals artifacted by the stimulation (see Figure S1 D). A Wilcoxon Signed-ranks Test indicated that the ‘residual’ level of gamma power within the 45-55 Hz band was higher when the artifact removal method was applied to an iEEG signal artifacted with real stimulation [*W* = 2006, *p* <.0001].


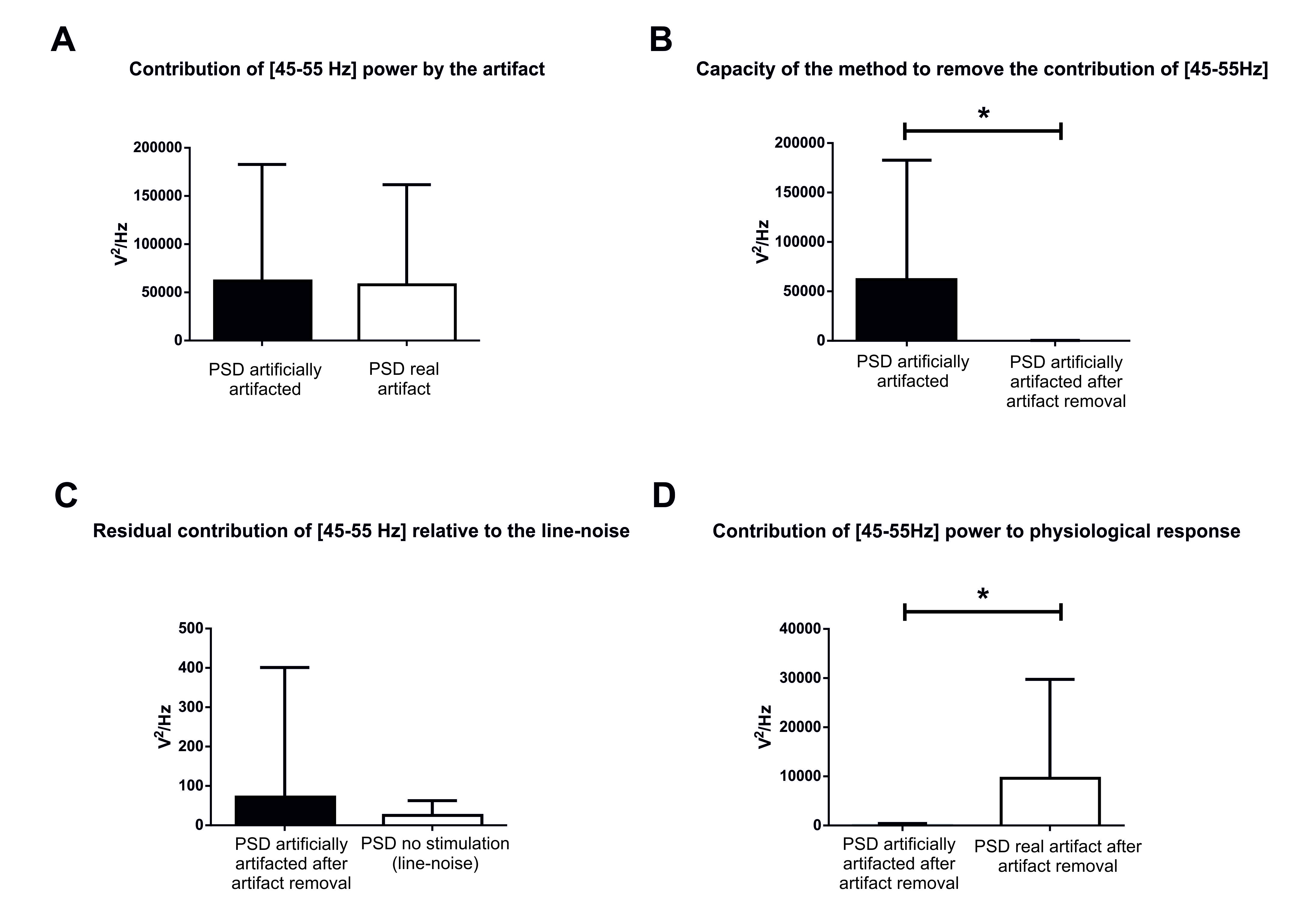


Figure S2. Ability of our artifact removal method to cancel stimulation artifacts in the [45-55Hz] gamma band. Bars represent the mean power spectral density (PSD in V^2^/Hz). whereas vertical lines represent the standard deviation. A. Comparison of the amount of gamma [45-55Hz] power in *artificially artifacted* iEEG signals with gamma [45-55 Hz] power present in iEEG signals during real 50 Hz electrical stimulation. B. Ability of our artifact removal method to reduce gamma [45-55Hz] power activity. C. Comparison between baseline gamma [45-55 Hz] power activity present in the signal after applying our artifact removal procedure and line-noise present in non-artifacted iEEG data. D. Comparison between residual gamma [45-55 Hz] power activity after the application of our artifact removal method to artificially artifacted signals and to real artifacted iEEG signals. * *p* <.0001

1. **Generation of Surrogate data for the S-PLV assessment**

As reported by Hurtado and colleagues^1^ a meaningful use of phase locking requires knowledge of the distribution under the ‘null’ hypothesis for each independent pair of time-series with oscillatory activity. Since our analyses were essentially single-trial based, we were unable to shuffle trials and determine the null distribution. To overcome this difficulty, we built for each dataset (i.e., the iEEG trace from each individual contact of a given multielectrode) a large ensemble of surrogate time series (n=30) following the method suggested by Hurtado and colleagues^1^ *(*see details in these author’s paper^1^ under the section *“SURROGATE METHOD S3: Surrogates that preserve the power spectrum of the instantaneous frequency*”). The set of surrogate time series generated using this method kept the same power spectrum (as the time series of the instantaneous frequency associated to the original iEEG data). The procedure randomized the instantaneous phase, while preserving the amplitude spectrum, with the restriction that negative and positive phase values remained symmetrical. Then, a distribution of single trial phase locking values was generated using these set of surrogate data and the signal corresponding to the stimulation output. The surrogate test consisted in comparing statistically, whether the stability of the phase locking value (S-PLV) measured in the original iEEG data was higher than the distribution of S-PLV values obtained in the surrogate data. When a given time series did not fulfill this condition, the measured S-PLV was not included in the analyses reported in our manuscript. Note that only 1.2% of the iEEG traces (n=14) did not pass the surrogate test, hence were not included in further analyses.

**REFERENCES**

1. Hurtado, J. M., Rubchinsky, L. L. & Sigvardt, K. A. Statistical Method for Detection of Phase-Locking Episodes in Neural Oscillations. *J. Neurophysiol.* **91**, 1883-1898 (2004).
